# Proton Pump Inhibition Increases Rapid Eye Movement Sleep in the Rat

**DOI:** 10.1155/2014/162314

**Published:** 2014-02-19

**Authors:** Munazah Fazal Qureshi, Sushil K. Jha

**Affiliations:** School of Life Sciences, Jawaharlal Nehru University, New Delhi 110067, India

## Abstract

Increased bodily CO_2_ concentration alters cellular pH as well as sleep. The proton pump, which plays an important role in the homeostatic regulation of cellular pH, therefore, may modulate sleep. We investigated the effects of the proton pump inhibitor “lansoprazole” on sleep-wakefulness. Male Wistar rats were surgically prepared for chronic polysomnographic recordings. Two different doses of lansoprazole (low: 1 mg/kg; high: 10 mg/kg) were injected intraperitoneally in the same animal (*n* = 7) and sleep-wakefulness was recorded for 6 hrs. The changes in sleep-wakefulness were compared statistically. Percent REM sleep amount in the vehicle and lansoprazole low dose groups was 9.26 ± 1.03 and 9.09 ± 0.54, respectively, which increased significantly in the lansoprazole high dose group by 31.75% (from vehicle) and 34.21% (from low dose). Also, REM sleep episode numbers significantly increased in lansoprazole high dose group. Further, the sodium-hydrogen exchanger blocker “amiloride” (10 mg/kg; i.p.) (*n* = 5) did not alter sleep-wake architecture. Our results suggest that the proton pump plays an important role in REM sleep modulation and supports our view that REM sleep might act as a sentinel to help maintain normal CO_2_ level for unperturbed sleep.

## 1. Introduction

The proton pump helps in performing several important cellular functions including the homeostatic regulation of cellular pH and acidification of intracellular organelles [1]. It plays a critical role in the acid-base balance [2, 3] and its altered activity is associated with the development of pathophysiological conditions such as gastroesophageal reflex disease (GERD), increased osteoclast activity, increased amyloid beta production in the brain, reduction in brain acetylcholinesterase activity, and alteration in spatial and chromatic visual perception [4–11]. It is also intricately related to neurotransmitter release and their reuptake mechanisms [7]. Thereby, the proton pump may play an important role in the modulation of various physiological functions.

Patients with medical condition such as GERD also suffer from sleep disturbances and, if treated with the proton pump inhibitor (PPI), their sleep efficiency improves [6]. It is nevertheless not known, if the PPI can itself modulate the neuronal circuitries underlying sleep. The two sleep stages: nonrapid eye movement (NREM) and rapid eye movement (REM) sleep, are regulated by the hypothalamic and brainstem neural circuitries of the brain, respectively [12]. The brainstem locus coeruleus (LC) area has one distinctive group of neurons called “REM-OFF” neurons, which do not fire during REM sleep [12, 13]. It is believed that the complete cessation of firing of these neurons plays a permissive role in REM sleep generation [12]. The LC neurons also act as a chemosensor and play an important role in regulating the proton ion concentration by modulating the cardiorespiratory machinery [14, 15]. Interestingly, it has been observed that a slight increase in bodily CO_2_ concentration increases sleep with a moderate increase in REM sleep episode number [15–18], suggesting that sleep can be affected by the alteration in bodily CO_2_ concentration. Increased CO_2_ concentration contributes in decreasing the cellular pH (increase in proton ion concentration) [19] and the proton pump helps in maintaining a normal cellular proton ion concentration [1]. The PPI can perturb pump activity and ultimately may impair the acid-base homeostatic regulation [2]. Further, it has been reported that CO_2_ regulates the tonic activity of the LC neurons [20], the area which also modulates REM sleep [12]. Hence, it is possible that the PPI can also modulate REM sleep.

The change in intracellular pH is a major part of the intracellular signaling pathway, which alters the firing rate of LC neurons [21]. A few studies have demonstrated an activity-dependent acidification of neuronal cytoplasm, which is soon followed by the spontaneous process of alkalization [7, 22]. This biphasic pH change is achieved through the proton pump by extruding hydrogen ions [22], and if this biphasic pH change is perturbed by using a PPI, neuronal firing is inhibited [7]. The sodium-hydrogen exchanger (NHE), on the other hand, plays a role in the intracellular pH recovery [23]. Neurons maintain the homeostatic balance of their intracellular pH by means of both, the proton pump and NHE, but through different mechanisms [7, 22–25]. It has been observed that the brainstem NHE is upregulated by chronic acidosis but not by prolonged hypercapnia [26], whereas the LC neurons may be hyperpolarized during sustained hypercapnia [14]. As it is widely known that the LC neurons also play an important role in the modulation of REM sleep, therefore, we reason that the proton pump would also modulate REM sleep, but the NHE would not. Hence, we aimed to investigate the effects of the PPI “lansoprazole” and NHE blocker “amiloride” on sleep-wakefulness (S-W) in the rat.

## 2. Materials and Methods

In this study, we used male Wistar rats (300–350 gm). Rats were obtained from the university's animal house facility and brought to the school's in-house animal facility a week before the commencement of the experiments. Animals were maintained on a 12-hour light-dark (L : D) cycle (lights on at 7:00 AM) at 23-24°C room temperature. They were given food and water *ad libitum*. All procedures used in this study were approved by the Institution's Animal Ethical Committee (IAEC) of Jawaharlal Nehru University, New Delhi, India.

### 2.1. Surgical Procedures for Polysomnographic Recordings

Rats were surgically prepared for chronic sleep-wake (S-W) recordings. Surgery was performed in a sterile condition. The animal was anesthetized with isoflurane (0.25%) using anesthesia face mask. The head was shaved and then fixed in the stereotaxic instrument. A midline incision was made and the skin was cut aside to expose the skull. Two pairs of small, stainless-steel screw electrodes to record frontofrontal and parietoparietal electroencephalogram (EEG) were fixed bilaterally (2 mm lateral from the midline) over the frontal (anterior-posterior (AP) +2 mm; lateral (L) 2 mm) and parietal (AP: −2 mm, L: 2 mm) (reference from the bregma) cortices on the skull. One screw electrode was fixed in the nasal bone as a reference electrode. Three electrodes (flexible insulated wire except at the tip) were implanted in the dorsal neck muscles to record electromyogram (EMG) (third EMG was implanted as a safeguard). Free ends of EEG, EMG, and reference electrodes were connected to a 9-pin miniature connector, which was cemented onto the skull with dental acrylic and the neck skin was sutured. After surgery, the anaesthesia face mask was removed and the animal was taken out from stereotaxic instrument.

Postoperatively, the animal was treated with dexamethasone (1.5 mg/kg, i.p.) and nebasulf powder (antibiotic) for 3-4 days to control brain inflammation and infection. Soft food was given to the animal during the recovery period. The condition of the animals was monitored regularly during the postoperative recovery period.

### 2.2. Polysomnographic Recording Procedures

After one week of recovery from surgery, animals were habituated in the recording cage (white plexiglass of 12′′ × 12′′ × 11′′ length, width, and height). The recording cage was placed in a well-ventilated, sound and light dampened (black color plexiglass) recording chamber (48′′ × 24′′ × 24′′) to minimize external disturbances during the experiments. The recording chamber was illuminated with 20 Lux light. Food and water bottle were placed in the food cup and bottle holder attached to the recording cage. Animals were tethered with the recording cable through a commutator and habituated to the recording chamber for 6 hrs (11:30 AM–5:30 PM) on two consecutive days. Also, during this period, the EEG and EMG signals were examined in a computer through Spike2 software (Cambridge Electronic Design, Cambridge, UK). EEGs were recorded in two channels and EMG was recorded in a single channel. Electrophysiological signals were amplified using 15LT Bipolar Portable Physiodata Amplifier System (Astro-Med, USA). EEG signals were processed with a high-pass filter of 0.1 Hz and a low-pass filter of 40 Hz, while EMG signal was processed with a high-pass filter of 10 Hz and a low-pass filter of 90 Hz digitized at 100 Hz sampling rate. Recordings were acquired in a personal computer using Spike2 software (Cambridge Electronic Design, Cambridge, UK) and were saved for offline analysis.

### 2.3. Experimental Design and Drugs Used

We investigated the effects of lansoprazole (PPI) and amiloride (NHE blocker) on S-W architecture in the rat. The surgically prepared animals for chronic S-W recordings were randomly divided into two groups: (a) lansoprazole group and (b) amiloride group. In the lansoprazole group, two different doses of the drug were injected in the same animal (*n* = 7) in a random order on two different days with a gap of at least one day. In the amiloride group, however, two different doses of the drug were injected in two different groups (low dose group: 10 mg/kg wt (*n* = 5), and high dose group: 50 mg/kg wt (*n* = 4)). Lansoprazole was dissolved in 200 *μ*L of dimethylsulfoxide (DMSO) and was diluted by adding 200 *μ*L of sterile 0.9% saline. Amiloride was also dissolved in 200 *μ*L of DMSO but was diluted with 200 *μ*L of sterile distilled water as it was precipitating in normal saline. Both of the drugs were injected in a volume of 400 *μ*L in all animals.

First, two baseline S-W data were recorded for 6 hrs (11:30 AM–5:30 PM) on two consecutive days (day 1 and day 2) in all groups after two days habituation in the same sleep recording chamber. Thereafter, in the lansoprazole group (*n* = 7), either of the two doses of lansoprazole (low dose (1 mg/kg) or high dose (10 mg/kg)) was injected intraperitoneally (i.p.), randomly on day 3 and the alternative dose was injected on day 5. Four hundred microliter vehicle (DMSO + normal saline 200 *μ*L each) was injected randomly either on day 4 or day 6.

In the amiloride group, however, 10 mg/kg and 50 mg/kg doses were used as low (*n* = 5) and high (*n* = 4) dose, respectively. Both of the dosages of amiloride were dissolved in 400 *μ*L of DMSO and water (with a ratio of 1 : 1) and were injected intraperitoneally. In these sets of experiments, low and high doses of amiloride were injected in two individual groups (for the reason that the animals treated with high dose of amiloride (*n* = 4) died 24–36 hours later). In the high dose group, S-W was recorded as baseline on day 1 and day 2, thereafter vehicle and high dose of amiloride were injected on day 3 and day 4, respectively. In the low dose group, however, the vehicle and drug were injected randomly either on day 3 or day 4 after two days baseline S-W recordings. The drugs and vehicle were injected few minutes prior to 11:30 a.m. and S-W was recorded for 6 hrs with baseline time matched hours soon after the injections.

### 2.4. Data Analysis

The polysomnographic records in Spike2 were converted into “European Data Format” and were scored offline using “Somnologica Science Software” (Medcare Flaga, Iceland). Records were displayed on a computer in “Somnologica” software and vigilant states were manually scored using 4 sec epochs employing the standard criteria for the rat. Low voltage and high frequency EEG waves associated with increased motor activity were analysed as wake; high voltage, low frequency EEG waves with prominent delta waves (0.5–4 Hz) and decreased motor activity were analysed as NREM sleep, whereas low voltage, high frequency EEG waves with a prominent theta peak (5–9 Hz) and nuchal muscle atonia were analysed as REM sleep. The total time spent in wake, NREM and REM sleep was calculated and was expressed as the total mean percent, every three hourly mean percent, and hourly mean percent out of the total recording time. Thereafter, changes in the amount of different vigilant states (total mean percent, three-hour mean percent, and hourly mean percent) were compared statistically between groups (baseline, vehicle, lansoprazole/amiloride low dose, and lansoprazole/amiloride high dose groups) using one-way repeated measures ANOVA followed by Tukey post hoc test. In the high dose of amiloride group, we have taken the data of only three animals (sleep data of the 4th animal could not be obtained after high dose of amiloride injection). The 50 mg/kg dose caused acute ionic stress and all animals died within 24–36 hours after amiloride injection. Therefore, we finally terminated the experiments of high dose of amiloride. After completion of experiments, animals were sacrificed with an overdose of cocktail anesthesia (80 mg/kg ketamine and 40 mg/kg xylazine) and the dead body was incinerated.

## 3. Results

### 3.1. Effects of the Proton Pump Inhibitor Lansoprazole on Vigilant States

**Figure 1:**
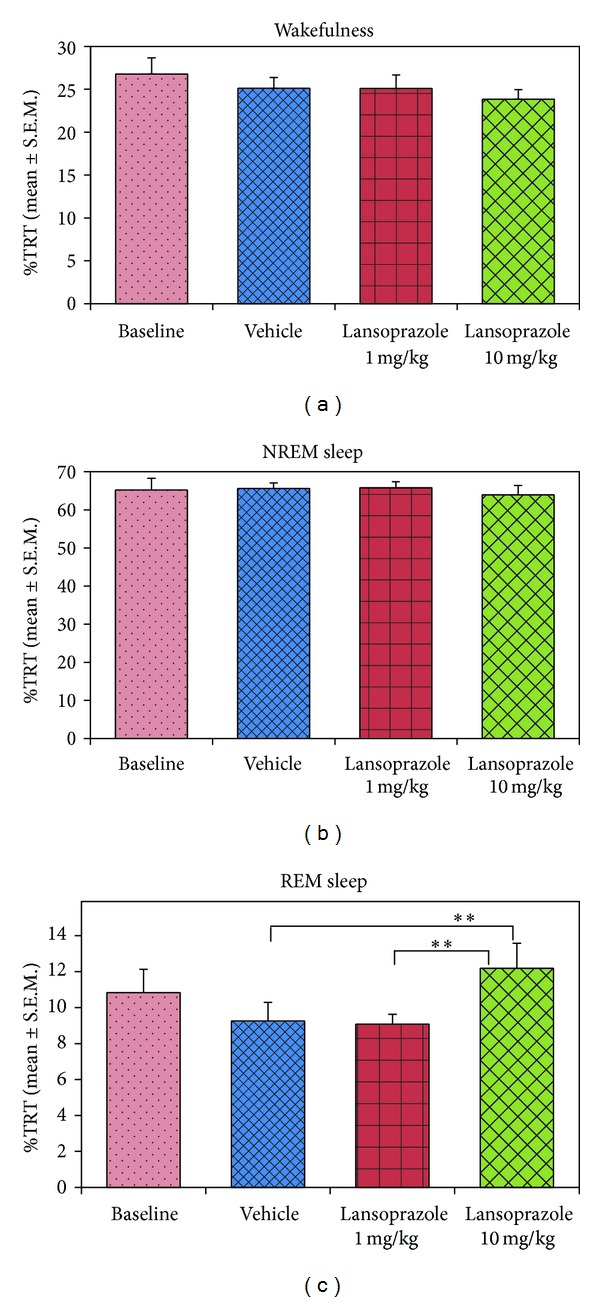
Percent wakefulness, NREM and REM sleep amount in baseline, vehicle, lansoprazole (low dose), and lansoprazole (high dose) out of total recording time (TRT). Lansoprazole (both low and high dose) did not increase (a) wakefulness and (b) NREM sleep, significantly. However, (c) REM sleep amount significantly increased after high dose (10 mg/kg) of lansoprazole injection (*P* < 0.01; *F*_(3,27)_ = 6.53) (one-way RM-ANOVA with Tukey post hoc tests) compared to vehicle (Tukey, *P* < 0.01) and low dose (1 mg/kg) lansoprazole (Tukey, *P* < 0.01) injections (i.p.). ** indicates *P* < 0.01.

**Figure 2:**
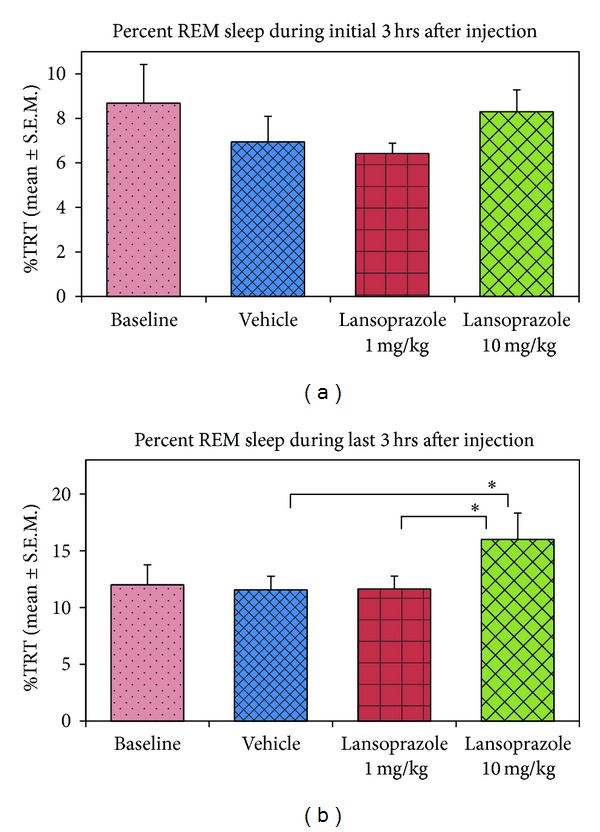
Percent REM sleep amount in baseline, vehicle, lansoprazole (low dose), and lansoprazole (high dose) groups during initial and later three hours of total recording time (TRT). (a) REM sleep amount did not change during the initial three-hour period after injection (*P* = 0.24; *F*_(3,27)_ = 1.52) (one-way RM-ANOVA with Tukey post hoc tests), (b) it significantly increased, however, after three hours of lansoprazole injection (*P* < 0.05; *F*_(3,27)_ = 4.07) (one-way RM-ANOVA with Tukey post hoc tests) compared to vehicle (Tukey, *P* < 0.05) and low dose (Tukey, *P* < 0.05). * indicates *P* < 0.05.

**Figure 3:**
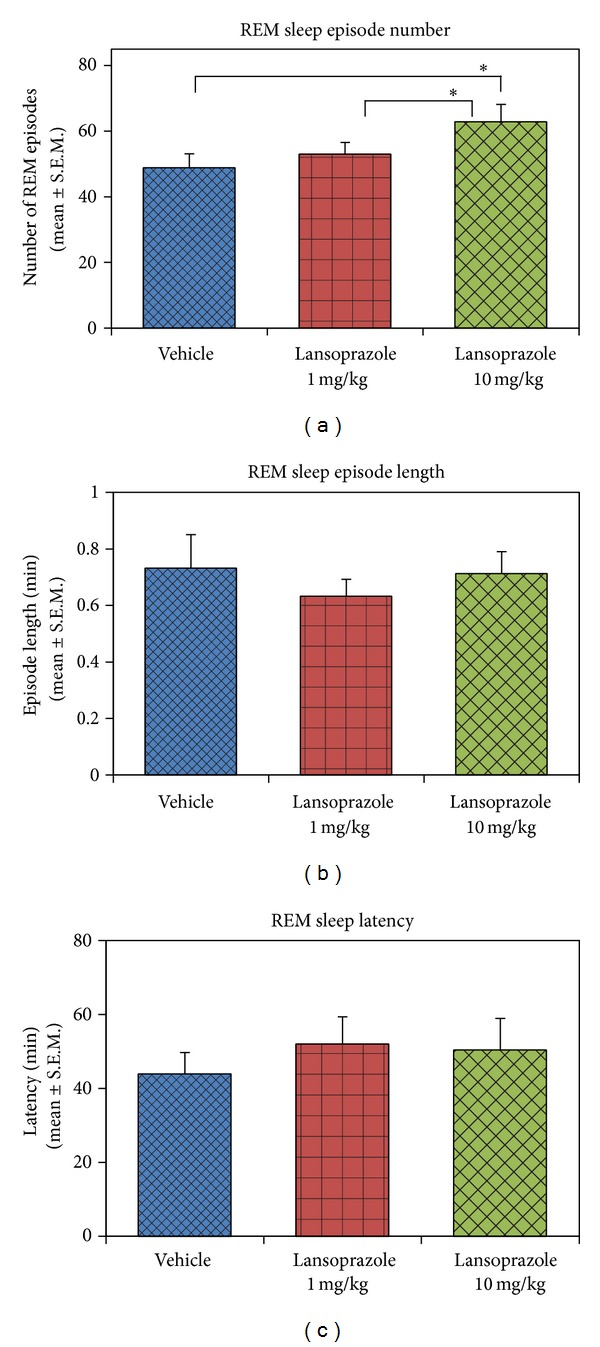
REM sleep episode number, episode length and latency in vehicle, and lansoprazole low and high dose treated animals. (a) REM sleep episode number significantly increased in lansoprazole high dose (10 mg/kg) compared to vehicle and lansoprazole low dose (1 mg/kg) treated rats (*P* < 0.05; *F*_(2,20)_ = 4.82). REM sleep episode duration length (b) and latency (c), however, did not change. * indicates *P* < 0.05.

Out of the total six-hour recording period, the high dose of lansoprazole (10 mg/kg) significantly increased REM sleep amount compared to the vehicle and low dose of lansoprazole (*P* < 0.01; *F*_(3,27)_ = 6.53). The amount of wakefulness (Figure 1(a)) and NREM sleep (%TRT), however, did not change (Figure 1(b)). Percent REM sleep amount in the animals of vehicle control and low dose (lansoprazole: 1 mg/kg) groups was 9.26 ± 1.03 and 9.09 ± 0.54, respectively, which increased significantly (*P* < 0.01; *F*_(3,27)_ = 6.53) by 31.75% (Tukey, *P* < 0.01) and 34.21% (Tukey, *P* < 0.01), respectively, in the animals of high dose group (Figure 1(c)). Animals in the baseline group exhibited 17.06% more REM sleep compared to the vehicle control animals. It was, however, not significant statistically (Figure 1(c)). Further, we observed in the hourly analysis that percent REM sleep showed a trend towards an increase in the high dose group at every hour starting from the 3rd to 6th hour time period compared to the baseline, vehicle, and low dose groups, with a statistically significant increase only at the 4th hour (*P* < 0.05; *F*_(3,27)_ = 3.88) (data not shown). Thereafter, we analyzed the changes in the REM sleep amount during the initial three hours and after three hours of lansoprazole injection. The percent REM sleep during the initial three hours period after lansoprazole injection did not change (*P* = 0.24; *F*_(3,27)_ = 1.52) (Figure 2(a)), but, interestingly, after three hours of injection, it increased significantly (*P* < 0.05; *F*_(3,27)_ = 4.07) compared to the vehicle (Tukey, *P* < 0.05) and lansoprazole low dose (Tukey, *P* < 0.05) (Figure 2(b)). Lansoprazole significantly increased REM sleep frequency only (*P* < 0.05, *F*_(2,20)_ = 4.87), while REM sleep episode duration length and REM sleep latency remained unaltered (Figure 3). Injection of high dose of lansoprazole did not induce any apparent behavioral or physiological changes such as restlessness or increased breathing rate in the animals. All animals exhibited normal cage behavior (normal eating and drinking behavior) and were active and inquisitive after injection. The experimenter closely observed the behavior of each animal after every drug injection.

### 3.2. Effects of the Sodium-Hydrogen Exchanger Inhibitor Amiloride on Vigilant States

Two different doses of amiloride: 50 mg/kg wt (*n* = 4) and 10 mg/kg wt (*n* = 5), were injected in rats. All animals treated with amiloride at 50 mg/kg died. One animal died after 24 hrs and three died 36 hrs later. These animals died possibly due to ionic stress and dehydration. Although 100 mg/kg dose has been used previously for an anticonvulsant effect in the rodent with no reports of animal casualty [27], however, this dose may cause intestinal perforation and peritonitis [28]. We could not check intestinal perforation or peritonitis but, to our observation, the animals injected with a high dose of amiloride (50 mg/kg) became sluggish and showed signs of dehydration 10–15 hours after injection. Skin became soft and flaccid and the pinched skin once released did not come down straight back soon (a clear sign of dehydration). Even supplementing saline or dextrose saline did not improve the condition of the animals and they ultimately died. Further, we noticed that the animals exhibited increased breathing rate and were urinating copiously. However, 6-hour S-W data from three animals (S-W data from one animal could not be collected after high dose of amiloride injection) exhibited that these animals were significantly more awake compared to the baseline and vehicle groups (percent wakefulness-baseline: 24.61 ± 0.39; vehicle: 24.04 ± 0.31; amiloride: 33.46 ± 2.65) (*P* < 0.05, *F*_(2,8)_ = 9.83) (compared to baseline Tukey, *P* < 0.05; compared to vehicle Tukey, *P* < 0.05). Animals spent significantly less time in NREM sleep compared to the baseline and vehicle groups (percent NREM sleep-baseline: 66.61 ± 0.81; vehicle: 66.45 ± 0.65; amiloride: 56.21 ± 3.63) (*P* < 0.05; *F*_(2,8)_ = 9.32) (compared to baseline Tukey, *P* < 0.05; compared to vehicle Tukey, *P* < 0.05). Percent REM sleep amount, however, was comparable (baseline: 8.78 ± 0.99; vehicle: 9.50 ± 0.44; amiloride: 10.33 ± 0.99) (*P* = 0.16; *F*_(2,8)_ = 3.06).

Low dose of amiloride (10 mg/kg) neither caused any casualty (*n* = 5) nor induced any apparent physiological changes in all animals. Animals were not stressed or dehydrated, rather were active, alert, and exhibited normal S-W patterns. Amounts of wakefulness, NREM sleep, and REM sleep in these animals did not change and were comparable to the baseline and vehicle groups ((percent wakefulness: baseline: 22.73 ± 0.88; vehicle: 26.75 ± 3.72; amiloride: 27.04 ± 3.45) (*P* = 0.29; *F*_(2,14)_ = 1.45); (percent NREM sleep: baseline: 66.88 ± 0.99; vehicle: 63.86 ± 3.08; amiloride: 64.31 ± 2.34) (*P* = 0.55; *F*_(2,14)_ = 0.63); (percent REM sleep: baseline: 10.38 ± 1.39; vehicle: 9.39 ± 0.75; amiloride: 8.65 ± 1.52) (*P* = 1.74; *F*_(2,14)_ = 2.19)).

## 4. Discussion

We examined the effects of the proton pump inhibitor “lansoprazole” and sodium-hydrogen exchanger blocker “amiloride” on S-W in the rat. High dose of lansoprazole injection (10 mg/kg) (i.p.) did not alter NREM sleep but REM sleep was increased significantly compared to the vehicle and low dose of lansoprazole (1 mg/kg) injections. This increase was significant only during the last three hours of recording. Although a trend of consistent increase in REM sleep continued throughout the recording period compared to the vehicle and low dose of lansoprazole, a significant increase was observed during the fourth hour of recording. Although it is not clear why lansoprazole was more effective in inducing REM sleep three hours after the injection, some studies have shown that lansoprazole effect is initiated around or after 4 hours of treatment [29]. In addition, lansoprazole significantly increased the REM sleep episode number, but REM sleep average duration length and its latency did not change. It has been reported earlier that mild hypercapnia significantly increased NREM sleep with an increasing trend of REM sleep frequency [16–18]. We did not, however, find any change in NREM sleep but the change in REM sleep frequency is consistent with earlier findings [15–18]. The NHE blocker “amiloride” (10 mg/kg), on the other hand, was ineffective in inducing any change in S-W architecture. These results suggest that the PPI, in addition to having several other pharmacological effects such as bactericidal and modulatory effect on acid secretion, and so forth, had a potency to induce REM sleep.

The NHE blocker “amiloride” (10 mg/kg) did not alter sleep architecture. Although, high dose (50 mg/kg) did significantly increase wakefulness and inhibit NREM sleep in three animals, but all animals died 36 hrs after injection. It has been reported earlier that 100 mg/kg amiloride not only enhances anticonvulsant action of several antiepileptic drugs [27] but may also cause intestinal perforation and peritonitis [28]. Therefore, we preferred 50 mg/kg as high dose, but unfortunately all animals died even at this dose. We, however, could not determine the actual cause of death of these animals (*n* = 4), but, to our observation, it could be attributed to acute dehydration. Amiloride also acts as the potassium-sparing diuretic [30] and all animals treated with 50 mg/kg amiloride were urinating copiously. The apparent signs of dehydration were noticed 10–15 hrs after injection in these animals. Animals could not recover even with a periodic supplement of saline/dextrose saline and ultimately died. The S-W data obtained from three animals exhibited a significant increase in wakefulness. We could not ascertain if the change in sleep architecture in animals treated with high dose (50 mg/kg) of amiloride was indeed due to the direct action of the drug or because of an associated acute stress induced in response to the drug. The low dose of amiloride (10 mg/kg) neither induced any physiological change nor altered sleep-wake architecture, suggesting that it may not have any direct modulating effect on sleep-wakefulness.

The proton pump and NHE play a role in the modulation of several physiological functions including chemoreception. The brainstem chemosensory neurons discern changes in brain pH and activate cardiorespiratory response to regulate pH [31]. The proton pump is located within neurons, whereas NHE is found on the membrane in several brainstem nuclei including the LC [14, 23–25]. The LC neurons are highly sensitive to changes in CO_2_/H^+^ ion concentration [14]. It has been reported that raising the concentration of CO_2_ in the inspired gas mixture, in anesthetized rats, results in rapid increase in the firing rate of LC neurons [32]. In contrast, an isolated *in vitro* study has demonstrated that a persistently increased CO_2_ concentration hyperpolarizes LC neurons (although elevated level of CO_2_ was beyond physiological limit) [14]. CO_2_-induced alteration in the firing of LC neurons depends partly on the presence of CO_2_ level outside the neurons. Nevertheless, it is also modulated, in part, by the chemoreceptors located within the LC neurons [20]. Some studies suggest that the proton pump plays an important role in neuronal firing as well as in neurotransmitter release through a biphasic pH change: a brief acidification followed by a prolonged alkalinization of cytoplasm. If this biphasic pH change is perturbed, it inhibits neuronal firing [7, 22]. It is known that the PPI “lansoprazole” crosses the blood brain barrier, affecting the intracellular machinery [33] and, therefore, may alter neuronal activity.

Besides the chemosensory neurons, the LC also contains a unique group of neurons called “REM-OFF” neurons. These neurons remain silent during REM sleep [13] and if the LC is electrically or pharmacologically activated, it does not allow REM sleep to occur [34, 35]. During NREM sleep, breathing rate slows down and as a result CO_2_ level increases [36]. We have proposed earlier that if the CO_2_ level increases excessively, it may induce wakefulness over NREM sleep, as high CO_2_ level activates the LC neurons [14], which fire mostly during wakefulness [13]. However, if the increased CO_2_ stays for a while, it may hyperpolarize LC neurons [14] and possibly hyperpolarize the REM-OFF neurons too. Since hyperpolarization of the LC's REM-OFF neurons facilitates REM sleep, hence, it is likely that a sustained moderate increase in CO_2_ level would facilitate REM sleep. The proton pump modulates neuronal firing through a biphasic pH change. It is possible that lansoprazole might impair such biphasic pH alteration by blocking the proton pump and the persistent intracellular acidic condition may cause hyperpolarization of the LC neurons including the REM-OFF cells, a condition that facilitates REM sleep [12]. If this would be the case, then one would expect that the microinjection of lansoprazole in the LC may facilitate REM sleep. In fact, we have observed that the microinjection of lansoprazole in the LC (2 and 10 mM) significantly induced REM sleep dose dependently [37]. In this study, we have injected the drug intraperitoneally; hence, the role of some other pathways or factors cannot be ruled out. For example, lansoprazole has a potency to induce anti-inflammatory action by inhibiting the production of proinflammatory cytokines [4], which also modulate LC neurons and sleep-wakefulness [38].

## 5. Conclusions

In summary, the brainstem's LC neurons play an important role in REM sleep regulation as well as in chemoreception. It is, however, not clearly known if some of the chemosensory neurons are also a part of the REM sleep executive machinery and this needs further experimental verification. Nevertheless, our data suggests that the chemosensory neurons either directly or indirectly may modulate the REM-OFF neurons. This supports our view that REM sleep might act as a sentinel to help maintain normal CO_2_ level for unperturbed sleep.
